# Evolutionary Approaches to Combat Antibiotic Resistance: Opportunities and Challenges for Precision Medicine

**DOI:** 10.3389/fimmu.2020.01938

**Published:** 2020-08-27

**Authors:** Matthias Merker, Leif Tueffers, Marie Vallier, Espen E. Groth, Lindsay Sonnenkalb, Daniel Unterweger, John F. Baines, Stefan Niemann, Hinrich Schulenburg

**Affiliations:** ^1^Molecular and Experimental Mycobacteriology, Research Center Borstel, Borstel, Germany; ^2^German Center for Infection Research (DZIF), Partner Site Borstel-Hamburg-Lübeck-Riems, Hamburg, Germany; ^3^Cluster of Excellence Precision Medicine in Chronic Inflammation, Kiel, Germany; ^4^Evolutionary Ecology and Genetics, Zoological Institute, Christian-Albrechts-Universität, Kiel, Germany; ^5^Section of Evolutionary Medicine, Institute for Experimental Medicine, Kiel University and Max Planck Institute for Evolutionary Biology, Plön, Germany; ^6^Department of Internal Medicine I, University Hospital Schleswig-Holstein, Kiel, Germany

**Keywords:** AMR, drug resistance, evolution, precision medicine, evolutionary medicine

## Abstract

The rise of antimicrobial resistance (AMR) in bacterial pathogens is acknowledged by the WHO as a major global health crisis. It is estimated that in 2050 annually up to 10 million people will die from infections with drug resistant pathogens if no efficient countermeasures are implemented. Evolution of pathogens lies at the core of this crisis, which enables rapid adaptation to the selective pressures imposed by antimicrobial usage in both medical treatment and agriculture, consequently promoting the spread of resistance genes or alleles in bacterial populations. Approaches developed in the field of Evolutionary Medicine attempt to exploit evolutionary insight into these adaptive processes, with the aim to improve diagnostics and the sustainability of antimicrobial therapy. Here, we review the concept of evolutionary trade-offs in the development of AMR as well as new therapeutic approaches and their impact on host-microbiome-pathogen interactions. We further discuss the possible translation of evolution-informed treatments into clinical practice, considering both the rapid cure of the individual patients and the prevention of AMR.

## Introduction

The evolution of antimicrobial resistance (AMR) in bacteria by mutation of the chromosome or horizontal gene transfer is a naturally occurring phenomenon that can be observed even in the absence of human interventions (1, 2). However, it is the widespread use of antibiotics for almost a century that led in the actual public health crisis. Indeed, numerous pathogens show an increase in their antimicrobial resistance (AMR) levels, often directed at multiple antibiotic drugs (i.e., multidrug resistance, MDR). Infections caused by MDR pathogens are difficult, if not impossible to treat with the most commonly used drugs, thereby requiring the application of less effective and/or more toxic regimens. By the year 2050, more patients are expected to die from infections with MDR pathogens (10 million/year) than from cancer today (8.2 million/year) (3). Current treatment practices, exemplified by proverbs such as “One size fits all” and “Hit hard and hit early” at least partially contributed to this scenario, and are unlikely to suffice for the challenges that medicine will face in the future (4).

**Figure 1:**
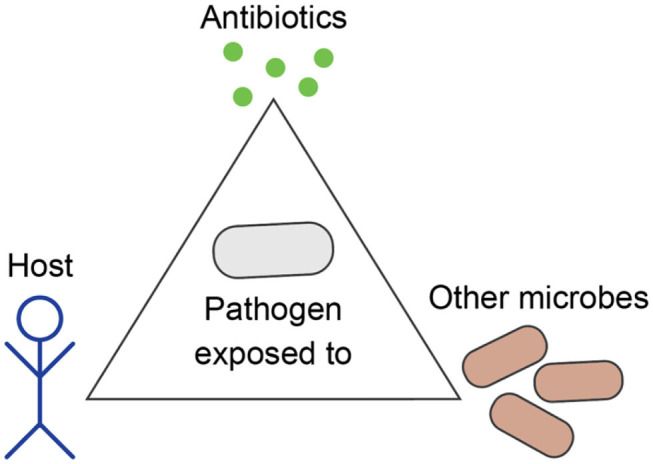
Evolution of bacterial pathogens during infection is influenced by the host's characteristics, other microbes, and antibiotics.

The field of Evolutionary Medicine strives to combat increasing drug resistance rates by exploiting evolutionary principles of resistance emergence and spread to design new treatment concepts (5, 6). The aims are 3-fold: (i) reducing intra-patient resistance selection that can lead to treatment failure, (ii) providing more rapid and less toxic cures to individual patients, and (iii) reducing the likelihood of AMR evolution and transmission at the population level. Achieving these aims is particularly challenging, because individualized patient care and public health considerations do not necessarily align. In particular, treatment regimens that were conventionally optimized to eliminate an infection as fast as possible impose a strong, often monotonic selective pressure on causal pathogens. Depending on bacterial adaptive capabilities, this can potentiate the risk for both short- and long-term resistance development (7). Furthermore, a broad activity of antimicrobials, which is often required for effective empirical anti-infective treatment, can compromise the healthy microbiome and thereby bears a risk for the development or aggravation of secondary diseases (8). Thus, we need to carefully consider evolutionary trajectories of pathogens in response to drug therapy, host immune system, as well as within a complex ecosystem of commensal microbial communities (Figure 1). In this context, Evolutionary Medicine is inevitably connected to applied Precision Medicine, where patients are stratified based on host, microbiome and pathogen characteristics, in order to deliver the most effective and sustainable treatment for a particular patient or group (9).

Here, we review (1) principles of bacterial resistance evolution and currently proposed concepts of evolution-informed therapies, (2) related challenges and opportunities, particularly with regard to host-microbiome-pathogen interactions, and (3) their relevance for clinical practice and Precision Medicine in the future.

## Principles of Drug Resistance and Evolution Informed Therapies

### Drug Resistance Mechanisms and Possible Fitness Costs

Throughout the course of evolution, molecules with antimicrobial activity were naturally selected in different organisms in order to outcompete bacteria in their environment by targeting essential functions such as DNA replication, transcription, translation, or metabolism (10). Although drug resistance evolution represents a paramount example of evolution observable in real-time, the design of antibiotic treatment regimens most often ignores the obtained insights into the involved selective processes (11). In fact, resistance evolving under antibiotic treatment in humans was traditionally seen as a phenomenon in individual patients (12, 13). Even if resistance-mediating adaptations arise in this context, their overall effect is assumed to be detrimental and changes may lead to lower fitness, so that resistant strains would usually not outcompete their susceptible counterparts (13). Thus, treatment designs over the past decades mostly focused on the principle of “Hit hard and hit early,” which goes back to the pioneering work of Paul Ehrlich from over 100 years ago (“*frapper fort et frapper vite*”) (14). Interestingly, Ehrlich already recognized the “great power of adaptation” and the risk for relapse and resistance evolution if a small number of pathogenic bacteria survives treatment (15). In fact, within the last decades, several pathogens acquired MDR, which prevailed under constant antibiotic selective pressure in hospitals and maintained transmissibility in the population (9).

One explanation for the success of AMR pathogens is the selection of low-cost or even no-cost resistance mediating mutations as shown for pathogens of the *Mycobacterium tuberculosis complex* (Mtbc), the causative agent of tuberculosis. The most prevalent mutations that mediate MDR, i.e., resistance against the widely used anti-tuberculous drugs isoniazid (katG p.S315T) and rifampicin (rpoB p.S450L), confer almost no fitness cost compared to wildtype strains (16, 17). Thus, even in the absence of the selective pressure of antibiotic therapy, MDR Mtbc strains persist and are associated with MDR tuberculosis outbreaks mainly in Eastern Europe, Central Asia, and South Africa (18, 19). Some resistance mechanisms, especially efflux pumps, are under extensive transcriptional control and can be amplified while antibiotics are present, so that any associated fitness costs are removed once resistance can be scaled back (20–22). Unlike Mtbc strains that evolve purely clonally, other bacteria such as *Pseudomonas aeruginosa*, an opportunistic pathogen in chronic lung infections, can acquire drug resistance not only by mutation, but also via horizontal gene transfer (HGT), e.g., involving plasmids (23). Such mobile resistance elements allow for the loss of AMR-mediating genes in the absence of antibiotic exposure, possibly attenuating AMR-associated fitness costs (24).

Another important evolutionary phenomenon observed in a number of AMR bacteria is compensatory evolution. Compensatory mutations are secondary mutations that reduce or ameliorate the fitness costs by the resistance mutation itself (25, 26) and have been described in a number of bacterial species *in vitro* (27, 28), and *in vivo* (28, 29), and have been associated with enhanced transmissibility (18, 19). Compensatory mutations can be clinically relevant, as they maintain resistance alleles and/or resistant clones in bacterial populations.

Beyond genetic mechanisms that allow bacteria to resist antibiotics or compensate for associated fitness costs, several species are able to express specific traits that protect them against antibiotics. *P. aeruginosa*, for example, forms biofilms and drug tolerant persister cells, leading to phenotypic resistance, often associated with chronic infection and relapse in patients with cystic fibrosis (30). *M. tuberculosis* has a unique thick cell wall and evolved a life cycle that mostly takes place in a granuloma (i.e., a concise structure of immune cells), which maintains the pathogen locally at the site of infection (e.g., the lung tissue) and shields from antibiotic penetration (31). Mtbc strains can also shift to a dormant state that reduces metabolic activity and increases the resistance to host/environmental stresses, including antibiotics (32).

These examples illustrate that drug resistance is a multifaceted evolutionary process, and that different pathogens have evolved diverse strategies which cause the traditional assumptions of antimicrobial therapy to fail. An in-depth understanding of these mechanisms may help to refine anti-infective therapies with currently available drugs, for example by focusing on antimicrobials, for which resistance comes at high costs (9).

From a public health perspective, resistance, and related bacterial fitness have to be extended to a broader context in which the (environmental) transmissibility of human pathogens and man-made selective pressures due to population-level antibiotic usage and its environmental distribution are considered (33). Moreover, the optimization of treatment strategies always has to be aligned with public health programs, in order to take account of insufficient disease control and surveillance measures as well as poor pharmaceutical control (e.g., availability of antibiotics in supermarkets), as observed in some structurally developing regions (34).

### Synergistic and Antagonistic Combination Treatments

MDR infections are often treated with drug combinations. Such combination therapy follows the rationale that the simultaneous emergence of resistance against employed drugs is less likely than that against single drugs. In detail, resistance against a hypothetical drug A (conferred by a mutation) may arise rapidly by chance, depending on the mutation frequency and the population size of the infecting pathogen. In contrast, the combined probability of the simultaneous occurrence of mutations conferring resistance against two different drugs A and B in any one cell is very low, not considering cross-resistance.

Combination therapy is often designed to enhance treatment efficacy by combining drugs that mutually enhance each other. A hypothetical drug pair A and B usually interact additively. Each drug individually may inhibit bacterial growth by 50% at a given concentration, in combination a theoretical growth reduction of 75% (residual growth = 0.5 × 0.5) is expected. The effect on growth can further be influenced by physiological interactions, so that antibiotic combinations become antagonistic (combination causes less growth reduction than expected based on the additive effect) or synergistic (combination leads to a higher growth reduction than expected) (35). Synergistic drug combinations that mutually improve their therapeutic effects should enhance treatment efficacy. Counterintuitively, however, several studies suggested that in the long-term they may rather promote the spread of resistance, possibly as a consequence of competitive release (i.e., the stronger effect eliminates competitors so that the surviving resistant variants obtain enhanced access to resources) (36–39).

Combination treatment may also select for mutations that confer resistance to both drugs simultaneously, instead of a sequence of individual drug resistances. De-repression of efflux pump expression is one example (40). While efflux pumps are generally upregulated under cellular stress, leading to temporary drug resistance, this upregulation can be made permanent through loss-of-function variants of repressor genes, as repeatedly observed in clinical isolates (41).

A recent systematic analysis of combination therapy in *P. aeruginosa* suggests that the picture is more complex. A comprehensive set of more than 1,600 evolution experiments and 38 distinct combination treatments demonstrates that synergistic antibiotic combinations increase the extinction of bacterial populations, even if drugs are used at sub-lethal level, while no clear effect on adaptation rates was observed in the surviving populations (42). Instead, evolutionary trade-offs appear to be the prime determinant of combination efficacy, in particular when these trade-offs take the form of evolved collateral sensitivities (see below), leading to significant reductions in adaptation rates, at least under the experimental conditions (42).

### Sequential Treatments

Sequential antibiotic treatment refers to the administration of different drugs in a chronological sequence to an individual patient, rather than their simultaneous combination, and in distinction to hospital drug rotation protocols (6).

Fundamentally, antibiotic monotherapy and sequential treatment both expose the patient and the infectious agent to one drug at a time. Evolutionary theory and *in vitro* experiments predict that bacteria are often able to adapt easily to a singular, continuous selective pressure (6, 43, 44). Consequently, *in vivo* observations confirm that resistance evolution indeed can happen within few days within patients (45). Antibiotic resistance phenotypes may be present at low frequencies in the infecting population or arise through spontaneous mutation, and subsequent selection by the antibiotic pressure (46). In a sequential treatment, however, selection of resistant subpopulations is repeatedly interrupted by changing the selective pressure. This strategy alone is able to slow the evolution of resistance down (47), and can be further amplified by limiting bacterial resistance evolution itself, through collateral sensitivity and negative hysteresis.

Collateral sensitivity (CS) was first described in the early antibiotic era (48), and has recently resurfaced as a promising strategy to limit bacterial resistance evolution (49). The effect results from an evolutionary trade-off, in which a mutation conferring resistance to one drug simultaneously causes increased sensitivity to another drug. The phenomenon of CS has now been described for a variety of bacterial taxa, such as *Acinetobacter baumannii, Eschericia coli, Enterococcus faecalis, P. aeruginosa*, or *Staphylococcus aureus*. It's strength and prevalence might depend however on the drug sequence, the bacterial species, and the evolutionary lineage within a bacterial species (44, 49–56). Overall, CS shows high potential for precision medicine to tailor treatments specific to a pathogen or even a specific strain (49, 57).

Whilst CS is based on changes in molecular structure (genetic mutations) occurring during resistance evolution, the concept of negative hysteresis (NH) results from a physiological response. In particular, NH occurs when the application of one antibiotic increases the efficacy of a second, subsequently applied antibiotic (58). Thus, increased susceptibility is not based on mutation and selection, but caused by physiological changes within a bacterial generation, even at sub-lethal antibiotic concentrations. NH may enhance the efficacy of sequential treatments, even at locations of poor drug penetration (e.g., biofilms) or with elevated levels of resistance (58).

## Host-Microbiome-Pathogen Interaction

In medical practice and research, pathogens are often studied as stand-alone entities that grow independently from other microbes and evolve antibiotic resistance as an adaptation to anti-infective therapy. However, pathogens are part of a complex ecosystem within the host, which can greatly influence the outcome of anti-infective treatment and the occurrence of AMR. During infection, pathogens evolve not only to resist antibiotics, but also to increase their fitness within the particular host in competition to the commensal microbiota, which needs to be taken into account when applying evolutionary approaches to medicine (Figure 1).

The spectrum of colonizing bacteria can be influenced by a variety of host mechanisms. For example, gastrointestinal glycans such as those generated by the enzyme encoded by the blood-group related gene *B4galnt2* were shown to be involved in shaping the resident microbiota (59) and, importantly, play a role in susceptibility to infection, e.g., by *Salmonella* (60).

Moreover, bacteria are forced to interact with other microbes upon colonization or infection of the host. In the process, commensal microbes impact the eco-evolutionary dynamics by affecting traits under selection (61). For example, mono-colonization of the mouse gut by *E. coli* can lead to predictable bacterial metabolic adaptations through the selection of mutations in genes related to amino acid metabolism, a process that is, however, altered by co-colonization with other microbes (61). Another example is colonization resistance, where presence of a complex residing microbial community can protect against pathogen infections (62).

Importantly, host-microbe and microbe-microbe interactions translate to ecological and evolutionary pressures that exhibit clinical relevance. These eco-evolutionary mechanisms affect both symbiotic organisms and pathogens, making their understanding key to sustainable pathogen control.

Furthermore, several known forms of bacterial interaction are directly linked to antimicrobial resistance.

### Bacterial Co-operation

During infection, pathogens can engage in cooperative behavior. For example, *P. aeruginosa* in the lungs of cystic fibrosis patients acquires iron via the siderophore pyoverdin, which is costly to produce. Pyoverdin is secreted into the bacterial environment where it represents a public good: Either it gets taken back up in its iron-loaded form by the secreting cell, thereby offsetting the cost of its production, or by a non-pyoverdin-producing bacterium, which thereby benefits from iron uptake without paying the cost of siderophore production (63–65). Resistance to β-lactam antibiotics can be mediated in a similar cooperative manner: secreted β-lactamases act as a public good by simultaneously protecting bacteria that do not produce the enzyme (66–68).

### Bacterial Competition

Bacteria-bacteria killing is a form of bacterial competition and can be mediated by the type VI secretion system (T6SS). In patients with *A. baumanii* infection, however, some bacterial isolates display an inactivated bacterial killing machinery whilst carrying several antimicrobial resistance genes (69). In these isolates, a large, self-transmissible plasmid encoded genes for resistance toward antibiotics ranging from beta-lactams to chloramphenicol, whilst carrying a negative regulator of T6SS. Only bacteria that could suppress their T6SS (thereby reducing elimination of surrounding bacteria) were able to successfully transmit the AMR-encoding plasmid containing the T6SS suppressor to other microbes, additionally facilitating the transmission of other AMR-encoding plasmids (70). Thus, a mechanistic linkage to bacteria-bacteria killing mechanisms can directly affect the spread of AMR.

### Bacterial Communities

Within an infected host, individual bacteria usually grow as part of communities with specific composition and spatial organization. In the example of *P. aeruginosa* in cystic fibrosis lungs, isolates from one particular site of a patient's lung can show resistance to a particular antibiotic, whereas bacteria from other sites of the same lung do not (71). Laboratory studies supported by computational modeling previously detected different levels of AMR depending on the density of the community and the levels of bacterial mixing (72, 73). As another example, *E. coli* may either tolerate higher concentrations of antibiotics (74) or, alternatively, be prevented to evolve resistance when embedded in a diverse bacterial community, highlighting the importance of bacterial communities for AMR of pathogens (75).

In a nutshell, bacterial pathogens are part of complex ecological environments, where their evolutionary dynamics are heavily affected by host-microbe and bacteria-bacteria interactions with cooperation, competition and community organization directly affecting resistance to antimicrobials. Looking ahead, the challenge will be to incorporate these aspects of a host's ecology and evolutionary history, and bacterial life into our understanding of AMR to inform therapy design.

## Relevance for Clinical Practice and Precision Medicine

Currently favored policies to counter AMR foremost focus on limiting the use of antibiotics to necessary medical treatments and encouraging the development of new antimicrobial substances (76). Whilst these measures are self-evident, they are not sufficient to provide a sustainable solution to the problem on their own. Evolutionary adaptation is at the very core of the bacterial AMR crisis. Therefore, the development of future treatment strategies against bacterial infections should necessarily follow evolutionary principles. Newly developed antimicrobial substances will at most provide a temporary relief, as evolution is a continuously ongoing process and almost certainly, resistance will eventually emerge against any new substance. Approaches following the “hit hard and hit early” principle, present since the beginning of the antibiotic era, need to be refined, taking into account individual characteristics of the infecting pathogen lineages, the affected patient, and the associated microbiota. Especially chronic recurring infections (such as *P. aeruginosa* in cystic fibrosis) or long-term antibiotic use (such as tuberculosis therapy) highlight that in numerous cases, due consideration should be given to avoid promoting resistance unintentionally by merely focusing on the simplicity of therapy. Even though evolution informed treatment strategies such as those outlined above might come with more complicated and time intensive protocols than those currently applied, they have the likely advantage to reduce the rate of resistance evolution and thereby improve therapy outcome, not only in individual patients, but also on the population level.

The complex interactions of pathogens with the host, local environments and residential microbiota render their evolutionary responses to antimicrobial substances when applied *in vivo* just as complex. This is where Precision Medicine comes into play. There is an urgent need to characterize bacterial infections, including identification of the pathogen taxa, the site of infection(s), phenotypic and genotypic drug resistance profiles, but also individual host and microbiome characteristics for better and more sustainable treatments. Developing and applying tailored treatments, however, comes with challenges inherent to Precision Medicine, requiring thoughtful balancing of individual and public interests: Deep sampling of pathogens in an infection for current explorative research as well as for future diagnostics or therapy surveillance, in conjunction with comprehensive characterization of patients in a system medicine approach comes with a high demand of personal and material resources. If patients are prone to undergo more intensive and invasive procedures than currently required when adhering to established treatment concepts, physicians may run into an ethical dilemma when weighing the benefit for the individual patient against the benefit for the public. However, evolution-informed therapies may offer an opportunity to meet both of these requirements by ensuring pathogen elimination and minimizing resistance spread, thereby sustaining treatment efficacy.

## Author Contributions

MM, LT, MV, EG, LS, and DU drafted the manuscript. MM, SN, and HS conceived the manuscript. All authors made intellectual contributions, critically revised the manuscript, and gave final approval.

## Conflict of Interest

The authors declare that the research was conducted in the absence of any commercial or financial relationships that could be construed as a potential conflict of interest.
